# Impact of Total Ischemic Time on Clinical Outcomes in Patients With ST-Elevation Myocardial Infarction: Lost Time Is Never Found Again

**DOI:** 10.7759/cureus.23143

**Published:** 2022-03-14

**Authors:** Pradeep Kurmi, Vishwa D Tripathi, Sunil K Tripathi

**Affiliations:** 1 Department of Cardiology, Super Speciality Block, Shyam Shah Medical College, Rewa, IND

**Keywords:** total ischemia time, st-elevation myocardial infarction, mortality, door to balloon time, angiographic findings

## Abstract

Introduction

A dedicated relationship between total ischemic time (TIT) and clinical outcomes has been reported in ST-elevation myocardial infarction (STEMI) patients undergoing percutaneous coronary intervention (PCI); however, this claim is yet to be clarified. Accordingly, this study was carried out to determine the association of TIT with in-hospital and one-year follow-up outcomes in STEMI patients undergoing primary PCI.

Material and methodology

Between December 2020 and December 2021, a total of 113 consecutive STEMI patients undergoing primary PCI were prospectively included. According to TIT, all patients were categorized into two groups: (a) shorter TIT (<180 minutes) and (b) prolonged TIT (≥180 minutes). Data regarding baseline, clinical, and angiographic characteristics, as well as in-hospital and one-year follow-up outcomes were noted among the two groups.

Results

A total of 113 STEMI patients with a mean age of 69.3 ± 13.6 years were studied, and males [92 (81.4%)] were predominately affected with STEMI. A median TIT was 348 minutes. Of 113, 30 (23.0%) patients had a TIT of <180 minutes and 83 (73.5%) had a TIT of ≥180 minutes. Prolonged ischemia duration was significantly associated with composite of death, rehospitalization, and revascularization (*p=*0.02) at one-year follow-up.

Conclusion

TIT can be considered a good quality indicator, together with door-to-balloon time and other clinical determinants, in order to improve survival in STEMI patients.

## Introduction

Myocardial infarction (MI) has reached epidemic proportions worldwide. Mortality from MI is swiftly increasing in developing countries, especially in India. The ST-Elevation Myocardial Infarction (STEMI) Programme initiated by the National Health Mission (NHM) in Tamil Nadu endorses 3,50,000 to 4,00,000 MI deaths every year in India [1]. Percutaneous coronary intervention (PCI) is the preferred reperfusion modality for STEMI when done in a timely fashion and by experienced operators. Globally, door-to-balloon (D2B) time has long been recognized as an integral quality indicator in the performance of hospitals catering to STEMI patients [2,3]. Recent initiatives dealing with STEMI management have focused on curtailing D2B time; in this regard, current joint clinical practice guidelines of the American College of Cardiology (ACC)/American Heart Association (AHA)/Society for Cardiovascular Angiography and Interventions (SCAI), and European Society of Cardiology (ESC) advocate D2B time to within 60 minutes after STEMI diagnosis [4]. This focus overlooks the vast number of STEMI deaths that occur before patients even arrive at the hospital. Moreover, it has been postulated that reduction in D2B time has no further benefits to STEMI patients who present late after the onset of symptoms [5]; thus, attempts to shorten D2B time have not been consistently associated with reduced mortality in STEMI patients [6]. With the establishment of the fact that total ischemic time (TIT) can be a more powerful prognostic indicator than D2B time [5], over the course of time, new research has paid attention to investigating the association between TIT and mortality. We, therefore, designed a prospective study to examine the association of TIT with clinical outcomes (i.e., in-hospital and one-year follow-up outcomes) in STEMI patients undergoing PCI.

## Materials and methods

Study design

During the period of December 2020 to December 2021, a prospective non-randomized observational study was carried out at Super Speciality Hospital, Shyam Shah Medical College, Rewa, Madhya Pradesh, India. Consecutive STEMI patients undergoing primary PCI were prospectively included. STEMI patients managed with thrombolytic therapy was the sole exclusion criterion. The study was approved by the Institutional Ethics Committee (Approval No: IES-SSMC-007) and performed in accordance with the guidelines of the Declaration of Helsinki. Written informed consent was obtained from each of the participants.

Data collection

Data regarding baseline and clinical characteristics were reported. Severity of STEMI was categorized according to Killip's heart failure (HF) classification: class I, no clinical signs of HF; class II, HF with rales in half of the lung field and with S3 gallop; class III, frank acute pulmonary edema; and class IV, cardiogenic shock, hypotension, and evidence of peripheral vasoconstriction such as cyanosis, oliguria, and diaphoresis [7]. All patients with a provisional diagnosis of STEMI were stratified into two groups based on TIT: shorter TIT (<180 minutes) and prolonged TIT (≥180 minutes). Patients of all age groups having different risk factors were analyzed. Treatments given during hospitalization and follow-up were optimized as per the standard institutional protocol. In-hospital outcomes were reported. The patients were followed-up on an outpatient department (OPD) basis and telephonically at one year.

Definitions

· ST-segment elevation myocardial infarction (STEMI): Angina or angina equivalent lasting for >20 minutes and ST-segment elevation of 1 mm in two contiguous leads, or new left bundle branch block, or true posterior MI with ST depression of 1 mm in two contiguous anterior leads.

· Total ischemic time (TIT): Time from pain onset to establishment of infarct-related artery flow.

· Cardiogenic shock: Sustained hypotension with systolic blood pressure <90 mmHg for at least 30 minutes, unresponsive to fluid administration and associated with features of tissue hypoperfusion.

· Coronary flow of the infarct-related artery: It was determined visually by the operator and categorized based on TIMI grading system on a scale of 0 to 3 both before and after PCI.

· Diabetes mellitus: Fasting blood sugar level of 126 mg/dL (7.0 mmol/L) or higher, HbA1c > 7, or on treatment.

· Dyslipidaemia: Fasting cholesterol >200 mg/dL or on treatment.

· Door-to-balloon (D2B) time: Time from first hospital arrival to the first attempt at reperfusion with any intracoronary device.

· Major bleeding: The occurrence of any of the following: intracranial bleeding, intraocular bleeding, retroperitoneal bleeding, access site hemorrhage requiring surgery or a radiological or interventional procedure, hematoma ≥ 5 cm in diameter at the puncture site, reduction in hemoglobin concentration of 4g/dL without an overt source of bleeding, reduction in hemoglobin concentration of >3g/dL with an overt source of bleeding, re-operation for bleeding, or use of any blood product transfusion.

· Procedural success of percutaneous coronary intervention (PCI): Achievement of vessel patency to residual stenosis < 30%.

· Re-infarction: Recurrent chest pain or ischemic equivalent symptoms lasting 30 minutes, and new electrocardiogram (ECG) changes consistent with re-infarction, and the next creatine kinase myocardial band (CK-MB) or creatine kinase (CK) level measured approximately 8-12 hours after the event was at least 50% above the previous level or >3 upper limits of normal, whichever was greater.

· Systemic hypertension: Systolic blood pressure of >140 mmHg and/or diastolic pressure of >90 mmHg, or on treatment.

Data analysis

Continuous data were explained by mean and standard deviation, and categorical variables were described as percentage and frequency. Student's t-test for independent samples was used to compare the mean between two groups, and the chi-square test or Fisher's exact test was used for comparison of categorical data. A p-value of <0.05 was taken to indicate statistical significance. Data analysis was carried out by using Statistical Package for the Social Sciences (SPSS) Version 20.0 (IBM Corp., Armonk, NY, USA).

## Results

A total of 113 consecutive STEMI patients who underwent primary PCI were studied. The cohort was predominantly male [92 (81.4%)], with a mean age of 69.3 ± 13.6 years. Median TIT and D2B time were 348 minutes and 45 minutes, respectively. A total of 51 (45.1%) were smokers, 52 (46.0%) were hypertensives, 44 (38.9%) were diabetic, nine (8.0%) had contrast-induced nephropathy. Previous history of percutaneous transluminal coronary angioplasty (PTCA)/coronary-artery bypass grafting (CABG) was found in seven (6.2%) patients. A statistically significant differences were found for the following baseline characteristics: male gender (p=0.04), smokers (p=0.05), hypertension (p=0.03), diabetes mellitus (p=0.04), contrast-induced nephropathy (p=0.02), Killip class I (p<0.01), Killip class II (p<0.01), Killip class IV (p=0.02), pain-to-door time (p=0.02), advanced age > 75 years (p=0.02), and hours from onset to balloon < 3 hours (p<0.02). Demographic and clinical characteristics stratified according to TIT are outlined in Table 1.

**Table 1:** Baseline demography and clinical characteristics stratified according to total ischemic time (TIT) Data are expressed as mean ± SD or median or n (%). *Statistically significant. PTCA, percutaneous transluminal coronary angioplasty; CABG, coronary artery bypass graft

Characteristics	Total (N = 113)	Total ischemic time		p-Value
		≤180 minutes (n = 30)	>180 minutes (n = 83)	
Mean age, years	69.3 ± 13.6	57.3 ± 12.4	60.1 ± 14	0.33
Male	92 (81.4%)	24 (80%)	68 (81.9%)	0.04*
Female	21 (18.6%)	6 (20%)	15 (18.1%)	0.11
Smokers	51 (45.1%)	13 (43.3%)	38 (45.7%)	0.05*
Hypertension	52 (46.0%)	17 (56.7%)	35 (42.2%)	0.03*
Diabetes mellitus	44 (38.9%)	14 (46.7%)	30 (36.1%)	0.04*
Contrast-induced nephropathy	9 (8.0%)	1 (3.3%)	8 (9.6%)	0.02*
Previous PTCA/CABG	7 (6.2%)	5 (16.7%)	2 (2.4%)	0.08
Killip class				
I	47 (41.6%)	18 (60.0%)	29 (34.9%)	<0.01*
II	43 (38.1%)	9 (30.0%)	34 (41.0%)	<0.01*
III	12 (10.6%)	2 (6.7%)	10 (12.0%)	0.40
IV	11 (9.7%)	2 (6.7%)	9 (10.8%)	0.02*
Mean ejection fraction	40.5 ± 8.4	42.9 ± 7.8	39.6 ± 8.4	0.26
Median pain-to-door time, minutes	300	60	420	0.02*
Median door-to-balloon time, minutes	45	47.5	45	0.55
Median total ischemia time, minutes	348	150	372	0.52
Advanced age > 75 years	16 (14.2%)	2 (6.7%)	14 (16.9%)	0.02*
Hours from onset to balloon				
<3 hours	30 (26.5%)	30 (100%)	-	0.02*
6-9 hours	29 (25.7%)	-	29 (34.9%)	-
9-12 hours	30 (26.5%)	-	30 (36.1%)	-
12-24 hours	24 (21.2%)	-	24 (28.9%)	-

Of 113 patients, 30 (23.0%) had a TIT of <180 minutes and 83 (73.5%) patients had a TIT of ≥180 minutes. The angiographic and procedural parameters stratified according to TIT are presented in Table 2. There was a statistically significant difference between prolonged TIT and shorter TIT for the following parameters: single-vessel disease (SVD) (p=0.02), double-vessel disease (DVD) (p=0.01), triple-vessel disease (TVD) (p=0.02), infarct-related artery location of left anterior descending artery (LAD) + first diagonal branch (D1) (p<0.01), right coronary artery (RCA) + posterior left ventricular branch (PLB) (p<0.01), stent length > 30 mm (p<0.01), use of intra-aortic balloon pump (IABP) (p=0.05), femoral route approach (p =0.05), direct thrombin inhibitor - bivalirudin (p =0.02), and thrombosuction (p=0.04) treatment. The prolongation of TIT in our study cohort might be due to patients’ failure to recognize cardiac symptoms and unavailability of a nearby facility.

**Table 2:** Coronary angiographic and procedural parameters stratified according to total ischemic time Data are expressed as n (%). *Statistically significant. SVD, single-vessel disease; DVD, double-vessel disease; TVD, triple-vessel disease; LAD, left anterior descending artery; D1, first diagonal branch; LCX, left circumflex artery; OM, obtuse marginal; RCA, right coronary artery; PLB, posterolateral artery or branch; IABP, intra-aortic balloon pump; GP, glycoprotein; DTI, direct thrombin inhibitor

Characteristics	Total (N = 113)	Total ischemic time		p-Value
		≤180 minutes (n = 30)	>180 minutes (n = 83)	
Coronary artery disease				
SVD	44 (38.9%)	14 (46.7%)	30 (36.1%)	0.02*
DVD	36 (31.9%)	11 (36.7%)	25 (30.1%)	0.01*
TVD	28 (24.8%)	6 (20%)	22 (26.5%)	0.02*
Infarct-related artery location				
LAD + D1	63 (55.8%)	17 (56.7%)	46 (55.4%)	<0.01*
LCX + OM	8 (7.1%)	1 (3.3%)	7 (8.4%)	0.22
RCA + PLB	42 (37.2%)	13 (43.3%)	29 (34.9%)	<0.01*
Use of bioresorbable stent				
Stent length > 30 mm	44 (38.9%)	11 (36.7%)	33 (39.8%)	<0.01*
IABP	17 (15%)	3 (10%)	14 (16.9%)	0.05*
Femoral route approach	110 (97.3%)	30 (100%)	80 (96.4%)	0.05*
Pre-dilatation	93 (82.3%)	23 (76.7%)	70 (84.3%)	0.25
Post-dilatation	90 (79.6%)	23 (76.7%)	67 (80.7%)	0.22
GP IIb/IIIa inhibitor	40 (35.4%)	15 (50%)	25 (30.1%)	0.34
DTI-bivalirudin	10 (8.8%)	2 (6.7%)	8 (9.6%)	0.02*
Thrombosuction	88 (77.9%)	28 (93.3%)	60 (72.3%)	0.04*

As depicted in Table 3, a significant association was not found between TIT and in-hospital outcomes in terms of all-cause death, cardiac death, emergent CABG, stroke, ventricular septal rupture, bleeding (hemoglobin drop > 3%), and composite of death, rehospitalization, and revascularization. At discharge, 100% patients received aspirin, 38.9% received clopidogrel, 40.7% received ticagrelor, 23% received prasugrel, 100% received statins, 55.8% received β-blockers, and 56.6% received angiotensin-converting enzyme (ACE) inhibitors.

**Table 3:** In-hospital outcomes stratified according to total ischemic time Data are expressed as n (%). CABG, coronary artery bypass graft

In-hospital outcomes	Total (N = 113)	Total ischemic time		p-Value
		≤180 minutes (n = 30)	>180 minutes (n = 83)	
All-cause death	8 (7.1%)	1 (3.3%)	7 (8.4%)	0.32
Cardiac death	3 (2.7%)	1 (3.3%)	2 (2.4%)	0.15
Emergent CABG	3 (2.7%)	1 (3.3%)	2 (2.4%)	0.81
Stroke	3 (2.7%)	1 (3.3%)	2 (2.4%)	0.81
Ventricular septal rupture	1 (0.9%)	0 (0.0%)	1 (1.2%)	0.53
Bleeding (hemoglobin drop > 3%)	28 (24.8%)	6 (20.0%)	22 (26.5%)	0.41
Composite of all outcomes (%)	46 (40.7%)	10 (33.3%)	36 (43.3%)	0.46

At one-year follow-up, prolonged TIT was significantly associated with composite of death, rehospitalization, AND revascularization (p<0.03). Drugs prescribed at discharge were as follows: aspirin in 98.2% of the cases, clopidogrel in 61.9%, prasugrel in 12.4%, ticagrelor in 13.3%, single antiplatelet in 11.5%, β-blockers in 50.4%, ACE inhibitor in 35.4%, and statins in 96.5% (Table 4).

**Table 4:** One-year follow-up outcomes stratified according to total ischemic time (TIT) Data are expressed as n (%). Note: excluding in-hospital mortality. *Statistically significant

One-year follow-up outcomes	Total (N = 113)	Total ischemic time		p-Value
		≤180 minutes (n = 30)	>180 minutes (n = 83)	
All-cause death	15 (13.3%)	2 (6.7%)	13 (15.7%)	0.21
Cardiac death	7 (6.2%)	1 (3.3%)	6 (7.2%)	0.20
Rehospitalization	9 (8.0%)	2 (6.7%)	7 (8.4%)	0.20
Stroke	0 (0.0%)	0 (0.0%)	0 (0.0%)	-
Revascularization	21 (15.8%)	2 (6.7%)	19 (22.8%)	0.09
Composite of death, rehospitalization, and revascularization	52 (46.0%)	7 (23.3%)	45 (54.2%)	0.007

## Discussion

In this prospective study, we endeavored to assess the association of TIT with clinical outcomes in STEMI patients undergoing PCI and observed that the mortality rates were higher with prolonged TIT during the hospital stay and at one-year follow-up, but the values were not statistically significant. The high mortality with prolongation of ischemic time may be due to the fact that infarct size significantly affects myocardial tissue and continues to damage with each passing second of ischemic time [8-11]. As a result, even with optimal reperfusion (primary PCI), prolonged ischemic time may result in increased mortality as well as decreased myocardial salvage [11-13]. In this study, there were two (2.4%) cardiac deaths with prolonged TIT during the hospital stay, and this value was 6 (7.2%) at one-year follow-up. This indicated that death due to arrhythmic cause was increased with the prolongation of TIT. Our half of the study population used β-blockers; thus, the present study threw light on the fact that β-blockers were not associated with a lower risk of death at any time point up to one year.

A recent study by Khalid et al. [13] advocated reduction of TIT as a limelight strategy in STEMI patients to reduce the risk of mortality. This finding had provided supporting evidence to a study conducted by De Luca et al. [12], wherein he reported that each 30-minute delay in angioplasty increased the risk of mortality at one year by 7.5% in STEMI patients. Doddipalli et al. [14] carried out research on 346 Indian STEMI patients who underwent primary PCI and demonstrated significantly higher mean TIT (8.0 ± 3.6 hours) among expired patients as compared to alive patients (6.2 ± 2.8 hours). Koifman et al. [15] analyzed data of STEMI patients from six contemporary Acute Coronary Syndrome Israeli Survey (ACSIS) registries 2000-2010 and concluded higher mortality rates for patients with prolonged TIT (>180 minutes) as compared to those with shorter ischemic time (<180 minutes) (8.7% vs 5.9%, log-rank p-value=0.003) at one-year follow-up. Ample of evidence has confirmed TIT as an independent predictor of short- and long-term mortality in patients with STEMI [5,12,16,17]. A growing body of evidence has supported the concept that TIT can be a better prognostic factor of mortality as opposed to D2B time [11,17,18]; hence, recent emerging trends show the way toward more utilization of TIT as a quality indicator than D2B time in predicting mortality. Given that D2B time shortening is associated with reduction in mortality after primary PCI [19], it should not be regarded as the sole quality indicator. As per the authors’ perspective, the remarkable reduction in mortality can be achieved with a shift in focus of point on shortening of TIT from D2B time. Collectively, the majority of research looking at the influence of TIT in STEMI management, including ours, concurred association of TIT with mortality.

A number of factors that play key role in the prolongation of TIT are unawareness and patients’ failure to recognize cardiac symptoms, and unavailability of attendants, transportation, finance, and nearby facility [13]. Reduction in TIT can be achieved by conducting various awareness initiatives such as building awareness among the population about early recognition of symptoms and gaining urgent accessibility of health care, establishment of a spoke-and-hub distribution model, use of the prehospital ECG, use the strategy of direct emergency physician activation of the catheterization laboratory without routine cardiology consultation, introduction of a single call system for activating the whole STEMI team, establishment of time fixation for STEMI team members so that they will be available to receive the patient within 20-30 minutes, quick clearance of elective cases during routine work hours, and allocation of dedicated night STEMI team on rotation [14].

Several potential limitations of the study should be acknowledged. First, this was a single-center and non-randomized study. Second, the huge differences in baseline characteristics between patients with short TIT and those with prolonged TIT might restrict the comparability of the groups. Third, the time of onset of chest pain was recorded based on the patient or attendant's recall; therefore, we could not exclude the impact of recall bias, which may have some over or understating. Last but not least, patients were not followed up for a longer duration of time; hence, the relationship between TIT and long-term clinical outcomes remains uncertain. Further multicenter studies with large sample sizes are warranted.

## Conclusions

Findings of the current study strongly support that TIT can be a good quality measure together with D2B time and other clinical determinants in order to improve survival in STEMI patients. Hence, attempts should be made to shorten TIT in order to enhance patients’ survival.
